# Neurotropic and modulatory effects of insulin-like growth factor II in *Aplysia*

**DOI:** 10.1038/s41598-019-50923-5

**Published:** 2019-10-07

**Authors:** Nikolay Vadimovich Kukushkin, Sidney Paulina Williams, Thomas James Carew

**Affiliations:** Center for Neural Science, New York University, 4 Washington Pl, New York, NY 10003 USA

**Keywords:** Molecular neuroscience, Insulin signalling, Long-term memory

## Abstract

Insulin-like growth factor II (IGF2) enhances memory in rodents via the mannose-6-phosphate receptor (M6PR), but the underlying mechanisms remain poorly understood. We found that human IGF2 produces an enhancement of both synaptic transmission and neurite outgrowth in the marine mollusk *Aplysia californica*. These findings were unexpected since *Aplysia* lack the mammal-specific affinity between insulin-like ligands and M6PR. Surprisingly, this effect was observed in parallel with a suppression of neuronal excitability in a well-understood circuit that supports several temporally and mechanistically distinct forms of memory in the defensive withdrawal reflex, suggesting functional coordination between excitability and memory formation. We hypothesize that these effects represent behavioral adaptations to feeding that are mediated by the endogenous *Aplysia* insulin-like system. Indeed, the exogenous application of a single recombinant insulin-like peptide cloned from the *Aplysia* CNS cDNA replicated both the enhancement of synaptic transmission, the reduction of excitability, and promoted clearance of glucose from the hemolymph, a hallmark of *bona fide* insulin action.

## Introduction

Insulin and related peptides are among the most conserved signaling molecules in the animal kingdom^1–3^. Together with the insulin receptor family, also highly conserved, these ligands form a variety of regulatory systems documented in animals as diverse as insects and mammals. In vertebrates, insulin-like receptors (ILRs) are subspecialized into (i) insulin receptors (InsR) and (ii) insulin-like growth factor receptors (IGFRs), matching the subspecialization of insulin-like peptides into insulin and insulin-like growth factors (IGFs). Classically, insulin and IGF systems have been viewed as related but functionally distinct: insulin was thought to control mainly energy homeostasis though the insulin receptor (InsR), while IGFs to control growth and proliferation through the IGF1 receptor (IGF1R)^4,5^. Until recently, various neurotropic effects were attributed to IGFs^6–11^ but not insulin, to which the brain was thought to be unresponsive. In fact, downstream effects of insulin and IGFs overlap extensively in both the metabolic and proliferative domains^12^. Both types of ligands share at least some affinity to both InsR, IGF1R and a hybrid InsR/IGF1R receptor, further blurring categorical boundaries between insulin and IGF signaling^13^. Insulin and IGF receptors split from a common ancestor only in the chordate lineage^14^ (see also Fig. S1), and have thus evolved as a unified system.

Perhaps the most striking neural effects for IGF2 were described in the rat, where Chen and colleagues found that this growth factor is critical for the formation of particular types of memory^13^. However, the downstream mechanisms of this IGF2 effect remain poorly understood. Most peripheral effects of IGF2 are attributed to IGF1R^12^. Surprisingly, in the rat, blocking IGF1R did not prevent the neurotropic effect of IGF2^13^. Instead, memory enhancement required a different transmembrane effector unrelated to insulin-like receptors. This protein, generally termed the cation-independent mannose-6-phosphate receptor (M6PR), displays multiple ligand affinities and in certain mammals is also known as the IGF2 receptor (IGF2R) owing of its evolutionarily novel affinity to IGF2^15–17^. M6PR/IGF2R is generally thought to negatively regulate IGF2 action by scavenging it away from IGF1R for lysosomal degradation, but it can also act as a signaling receptor^18–21^, potentially contributing to the effects of IGF2. Thus, the effects of IGF2 on mammalian neural function may be unrelated to its involvement in the insulin-like system. Alternatively, M6PR/IGF2R may modulate more conventional, InsR/IGF1R-dependent functions of IGF2 through a mechanism that remains to be established.

*Aplysia californica* is a marine gastropod mollusk that has served as a powerful model system in molecular and cellular studies of memory, providing simultaneous experimental access to neural phenomena ranging from defensive behaviors to signaling cascades^22^. Owing to *Aplysia*’s evolutionary distance from mammals, its M6PR naturally lacks affinity to IGF2, since this interaction is specifically associated with placental mammals^17,23–25^. *Aplysia* also lacks the distinction between InsRs and IGFRs^14^ (see also Figs S2 and S3). It therefore presents a unique opportunity to examine conserved insulin-like signaling that may underlie or interact with specifically mammalian neurotropic effects of IGF2.

One particularly well-characterized aspect of *Aplysia* behavior is defensive withdrawal of the gill, siphon, or tail upon mechanical stimulation^22,26–28^. Much of this response is controlled by a monosynaptic glutamatergic circuit consisting of a mechanosensory neuron (SN), either in the ventrocaudal sensory cluster of the pleural gangion^29^, or in the LE cluster of the abdominal ganglion^30^, and a motor neuron (MN). This reflex, and the underlying circuit, are subject to multiple forms of plasticity, including sensitization in response to noxious stimuli such as an electric shocks. In response to a training shock, there is a global release of 5HT in the Aplysia CNS^31^, resulting in heterosynaptic facilitation that relies largely, if not exclusively^32^, on presynaptic mechanisms^33–40^. Depending on the pattern of stimulation^41–49^, such stimuli can lead to short-term or long-term memory at the behavioral level. This system has been extensively studied at various levels of analysis ranging from intact animals to isolated SNs. It is well established, for example, that a single pulse of 5HT produces short-term facilitation in the SN-MN circuit lasting minutes, whereas five spaced pulses produce presynaptic cAMP/PKA-dependent long-term facilitation (LTF) lasting >24 h^50^. Using a simplified preparation consisting of pleural SNs cultured with or without a postsynaptic partner (L7 motoneuron)^40,51,52^, we show in the present study that IGF2 in *Aplysia* promotes long-term synaptic facilitation and neurite growth, but simultaneously (and unexpectedly) reduces neuronal excitability, a combination of effects that we propose is associated with the homeostatic functions of *Aplysia*’s insulin-like system.

## Results

### IGF2 promotes the induction of LTF when combined with 5HT

**Figure 1:**
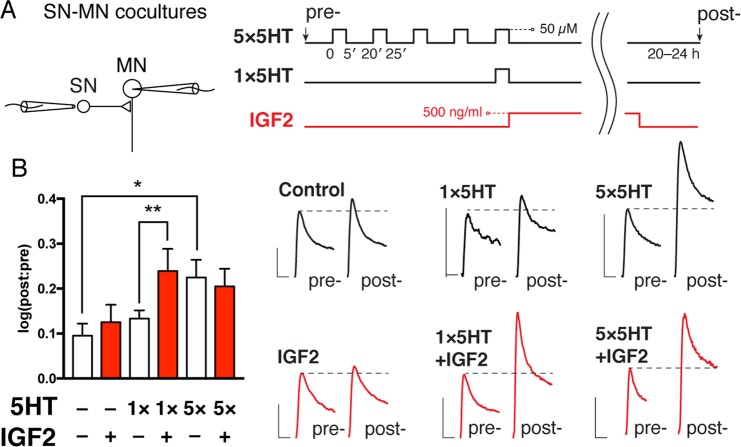
Gain-of-function effect of human IGF2 on *Aplysia* long-term facilitation. (**A**) LTF was recorded in cocultures of sensory and motor neurons. *Left*, sensory neurons were stimulated extracellularly to elicit a single action potential, and EPSP amplitudes measured in motor neurons by intracellular recording. *Right*, after pre-recordings cultures were subjected to one (1 × 5HT) or five (5 × 5HT) pulses of 5HT at 50 µM, each lasting 5 min (ITI = 15 min), followed by overnight incubation with or without IGF2 (500 ng/ml). Intracellular recordings were repeated 20–24 h after stimulation with 5HT. Untreated controls were included in each experiment and pooled with data shown in Fig. 3A (n = 21). (**B**) *Left*, changes in EPSP amplitudes were expressed as log10[post:pre]. *Right*, representative EPSP traces recorded for each condition before (pre-) and 20–24 h after (post-) treatment with 5HT. Scale bars, 5 mV/20 ms. Data are means ± SEM. Asterisks represent *p*-values in a two-tailed Student’s *t*-test. *p < 0.05.

We first asked the question: does human IGF2 affect LTF in *Aplysia*? To explore this issue, we treated cultured *Aplysia* SN-MN pairs with either one or five spaced pulses of 5HT, representing subthreshold and suprathreshold paradigms for inducing LTF^53^. Following the treatment, cells were incubated in full culture media (salt-adjusted L15 supplemented with 2 mM L-glutamine and 50% *Aplysia* hemolymph) in the presence or absence of IGF2 for 16–24 h. SNs were then stimulated to elicit a single action potential using an extracellular electrode, and PSPs were recorded intracellularly in MNs (Fig. 1). PSP amplitudes were measured before and after this treatment protocol, and changes expressed as log10 of the post:pre ratio. While 5 pulses of 5HT produced LTF (Control: 0.09531 ± 0.02671, n = 21; 5 × 5HT: 0.2249 ± 0.03937, n = 14; difference 0.1296 ± 0.04581, 95% CI 0.03639 to 0.2228; here and below data are presented as mean ± SEM), 1 pulse did not produce significant facilitation above untreated controls (1 × 5HT: 0.1332 ± 0.01849, n = 14). Addition of IGF2, however, resulted in significant synaptic facilitation when combined with a single pulse of 5HT (1 × 5HT + IGF2: 0.2391 ± 0.04936, n = 13; *p* = 0.008 in a two-tailed unpaired Student’s *t*-test; difference *vs* 1 × 5HT 0.106 ± 0.05124, 95% CI 0.0004641 to 0.2115), but not when applied to unstimulated cells (IGF2: 0.125 ± 0.03904). A combination of IGF2 with five pulses of 5HT did not significantly enhance synaptic transmission further (5 × 5HT + IGF2: 0.2049 ± 0.03938), suggesting occlusion of the IGF2 effects by repeated stimulation. Increased sample sizes for the control and IGF2 groups are because these conditions were included in each experiment, whereas other groups were randomized. To summarize, IGF2 displays a classic gain-of-function phenotype in the induction of *Aplysia* LTF.

### IGF2 induces neuronal growth, but reduces excitability

**Figure 2:**
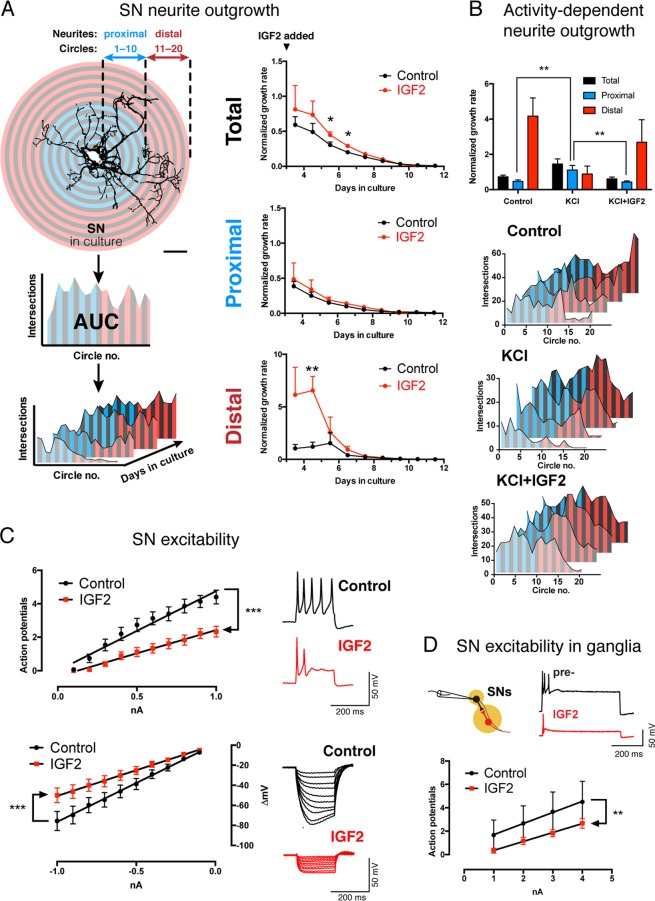
IGF2 promotes sensory neuron growth but reduces excitability. (**A**) IGF2 accelerates neurite outgrowth. Left, workflow for Sholl analysis. Neurons were imaged every 24 h following application of fresh culture medium with or without IGF2 3 d after plating. Number of neurite intersections with each of 20 concentric circles was plotted against the circle number, and areas under the resultant curve (AUC) calculated for each image. Growth rates per unit of AUC were calculated using either the total arbor in frame, or its proximal/distal parts. Scale bar, 100 µm. Right, changes in neurite growth rates over time in the presence of absence of IGF2, calculated using total, proximal or distal settings. *p < 0.05 in a two-tailed unpaired Student’s *t*-test. (**B**) IGF2 antagonizes a distinct activity-dependent form of growth. Following application of fresh culture medium 3 d after plating, neurons were exposed to three 5-min bouts of high-KCl (100 mM) salt water spaced by 2 h (+KCl), and their growth monitored over subsequent days with or without IGF2. *Top*, total, proximal and distal neurite growth rates calculated between DIV3–4 as in A. *Bottom*, representative Sholl profiles of neurons in different conditions over multiple days (see also Fig. S1). **p < 0.01 in Dunnett’s multiple-to-one post-hoc comparison. (**C**) IGF2 reduces excitability and input resistance of sensory neurons. Cells were exposed to IGF2 or vehicle 1 h prior to intracellular recordings. *Left*, I/O curves for positive (top) and negative (bottom) current injections showing number of action potentials fired, or membrane hyperpolarization elicited, respectively. ***p < 0.001 in ANCOVA comparing linear regression slopes. *Right*, representative responses to a 1 nA positive current injection (*upper right*), or 10 sweeps of negative current between 0.1–1 nA (*lower right*). (**D**) IGF2 reduces excitability of SNs in the pleural ganglion. SN excitability was measured in pleural ganglia by intracellular current injections before (pre-) and after treatment with IGF2. Data are means ± SEM. ***p < 0.001 in ANCOVA comparing linear regression slopes. Top, representative responses to a 3 nA positive current injection.

To further characterize the effects of IGF2 on *Aplysia* neurons, we monitored the morphology of cultured SNs by Sholl analysis over the course of multiple days (Fig. 2A). From these data, we calculated average neurite growth rates in the presence or absence of IGF2. IGF2 increased these rates and therefore stimulated neurite outgrowth (Fig. 2A), consistent with its LTF-promoting effects (Control: 0.304 ± 0.043 [arbitrary rate units], n = 7; IGF2: 0.4468 ± 0.04477, n = 10 between DIV 5–6; difference 0.1428 ± 0.06463, 95% CI 0.005081 to 0.2806; *p* = 0.043 in a two-tailed unpaired Student’s *t*-test; control: 0.2002 ± 0.02472, n = 7; IGF2: 0.2913 ± 0.02725, n = 10 between DIV 6–7; difference 0.09104 ± 0.0387, 95% CI 0.008562 to 0.1735; *p* = 0.033 in a two-tailed unpaired Student’s *t*-test).

An incidental observation over the course of these experiments led us to examine IGF2-mediated effects on growth enhancement in more detail. Averaged across the entire neuronal arbor, IGF2 increased neurite growth rates. Most of the growth rate increase, however, was concentrated in distal neurites (circles 11–20; control: 1.207 ± 0.4275 [arbitrary rate units], n = 6; IGF2: 6.555 ± 1.343, n = 8 between DIV 4–5; difference 5.348 ± 1.609; 95% CI 1.842 to 8.853, *p* = 0.006 in a two-tailed unpaired Student’s *t*-test; three cells were excluded because their distal neurites had not grown sufficiently by DIV4 to calculate growth rates). By contrast, in areas proximal to the soma there was little effect of the ligand (Fig. 2A). Curiously, this pattern was distinct from that induced by application of KCl at 100 mM (i.e. induction of neuronal activity), which is well known to promote growth^54–59^. In this latter case, much of the growth rate increase was confined to neurites most proximal to the soma, as opposed to the distal neurites (Fig. 2B and S1). Most surprisingly, when the KCl treatment was combined with IGF2, the enhancement of proximal neurite growth was abolished (circles 1–10; control: 0.4647 ± 0.0822 arbitrary rate units, n = 17; KCl: 1.114 ± 0.2526, n = 12; KCl + IGF2: 0.4253 ± 0.0672, n = 17). One-way ANOVA revealed a significant difference between means (*p* = 0.0017), Dunnett’s multiple comparisons test confirmed a significant increase of proximal neurite growth rates in the group treated with KCl alone compared to untreated controls and KCl + IGF2 cotreatment (KCl *vs* control difference 0.6496 arbitrary rate units, 95% CI 0.2038 to 1.095, *p* = 0.0034; KCl *vs* KCl + IGF2 difference 0.689, 95% CI 0.2433 to 1.135, *p* = 0.0019). Together, these data suggest that while both IGF2 and KCl promote neuronal growth, they do so by distinct mechanisms, and IGF2 can actually suppress the activity-dependent effects. The mechanisms underlying these distinct forms of growth, and their physiological significance, remain to be established. However, the observation that IGF2 counteracts the effects of KCl led us to ask whether IGF2 may have an effect on neuronal activity itself.

We therefore treated *Aplysia* SNs with human IGF2 and monitored their electrophysiological properties by intracellular recording coupled with current injections in bridge mode. For excitability measurements, we calculated the number of action potentials elicited by increasing amounts of positive current. For input resistance, we measured the hyperpolarization elicited by increasing amounts of negative current. We then performed linear regression and compared the slopes of the resulting I:O curves by ANCOVA. We found that IGF2 treatment for 1 h (n = 18) caused a reduction of excitability (Fig. 2C) and input resistance compared to untreated cells (n = 15) (excitability, control: 4.756 ± 0.3201 action potentials/mA positive current, R^2^ = 0.965; IGF2: 2.774 ± 0.1062, R^2^ = 0.9884, p < 0.0001; input resistance, control: 77.51 ± 1.076 mV/mA, R^2^ = 0.9985; IGF2: 51.36 ± 0.6921, R^2^ = 0.9985, *p* < 0.0001). This effect was sensitive to rapamycin, an inhibitor of mTOR and a highly conserved downstream signaling partner of insulin-like receptors (Fig. S5). To further examine this effect, we replicated the IGF2 treatment in intact pleural-pedal ganglia, which contain sensory neurons in an intact reflex circuit for defensive withdrawal (Fig. 2D). Similarly to cultured neurons, SNs in ganglia responded to IGF2 with significantly reduced excitability (0.95 ± 0.02887 action potentials/mA positive current, n = 6, R^2^ = 0.9982; IGF2: 0.7667 ± 0.02357, n = 6, R^2^ = 0.9981, *p* = 0.0079 by ANCOVA). Thus, IGF2 regulates the resistance of the neuronal membrane and reduces its excitability, which likely explains the blockage of KCl-induced effects on neuronal growth.

### A single endogenous insulin-like peptide reproduces neurotropic effects of IGF2 and promotes glucose absorption

How can the complex effects of IGF2 on *Aplysia* cells be interpreted from a functional perspective? It is important to consider that the evolutionary diversification of insulin and insulin-like growth factors, as well as the corresponding receptors from a single ancestral molecule, occurred within the chordate lineage^14^, and insulin-like receptors in *Aplysia* are therefore equally related to InsR and IGF1R (Figs S2 and S3). If IGF2 acts as a substitute for an endogenous factor, the role of that factor may be homeostatic control of metabolism and behavior in response to feeding, functions generally associated with insulin in mammalian systems. However, the alternative possibility is that IGF2 engages multiple independent signaling pathways that are not normally coordinated *in vivo*. Since the *Aplysia* insulin-like system includes multiple ligands and multiple receptors, it is possible that they are segregated into functionally independent modules engaged in distinct physiological situations. To test whether the combination of neural responses to IGF2 represents a naturally unified state, we cloned and expressed in *E*. *coli* one of five insulin-like peptides identified in the *Aplysia* genome and transcriptome by sequence similarity (ApILP1).

**Figure 3:**
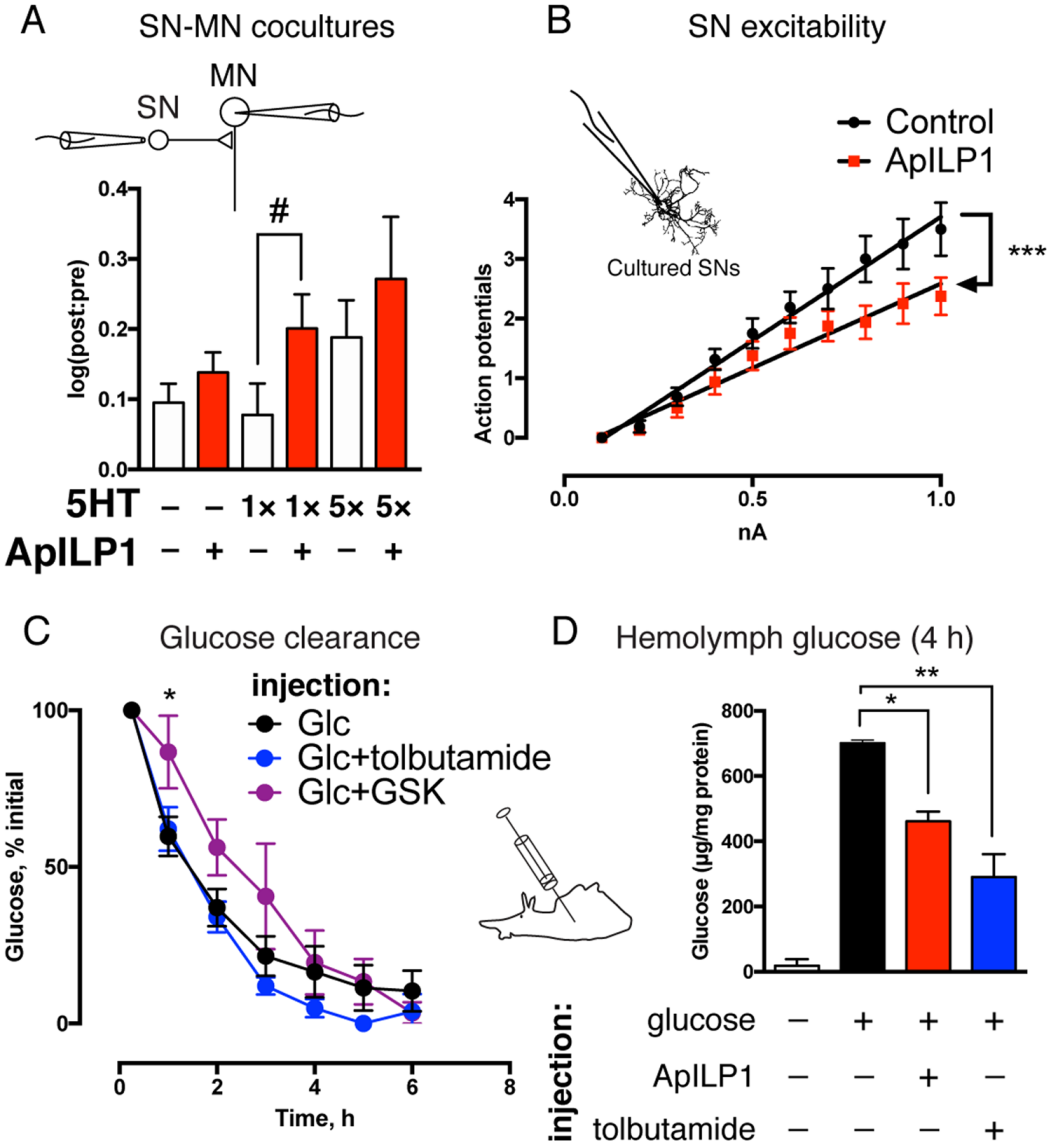
ApILP1 replicates neurotropic effects of IGF2 while promoting glucose absorption. (**A**) ApILP1 has a gain-of-function effect on LTF. LTF was recorded in cocultures of sensory and motor neurons as in Fig. 1. After pre-recordings cultures were subjected to one (1 × 5HT) or five (5 × 5HT) pulses of 5HT at 50 µM, each lasting 5 min (ITI = 15 min), followed by overnight incubation with or without ApILP1. Intracellular recordings were repeated 20–24 h after stimulation with 5HT. Changes in EPSP amplitudes were expressed as log10[post:pre]. ^#^p < 0.05 in a one-tailed unpaired Student’s *t*-test. Untreated controls were included in each experiment and pooled with data shown in Fig. 1A (n = 21). (**B**) ApILP1 reduces excitability of sensory neurons. Cells were exposed to ApILP1 or vehicle 1 h prior to intracellular recordings. Following injections of positive current, number of action potentials fired was counted. ***p < 0.001 in ANCOVA comparing linear regression slopes. (**C**) Endogenous clearance of glucose in Aplysia. 200–250 g animals were injected with a solution containing 100 mg glucose per 1 kg weight with or without tolbutamide or GSK1838705A (GSK), hemolymph samples collected at various time points, their glucose content measured and normalized to total protein concentration. Initial levels were measured 15 min post-injection and defined as 100%. *p < 0.05 in a two-tailed unpaired Student’s *t*-test (Glc *vs* Glc + GSK). (**D**) ApILP1 and tolbutamide accelerate glucose absorption in Aplysia. 1–4 g animals were injected with 500 mg glucose per 1 kg weight with or without tolbutamide or ApILP1, hemolymph collected 4 h after injection, glucose content measured as in C. Data are means ± SEM. *p < 0.05, **p < 0.01 in Dunnett’s multiple-to-one post-hoc comparison.

This peptide replicated the effects of IGF2 on synaptic facilitation (Fig. 3A) and SN excitability (Fig. 3B). Similarly to IGF2, it caused a gain-of-function effect on LTF when paired with a single pulse of 5HT (1 × 5HT: 0.07771 ± 0.04468 [log10(post:pre)], n = 17; 1 × 5HT + ApILP1: 0.2007 ± 0.04884, n = 13; difference 0.123 ± 0.06661; 95% CI –0.0134 to 0.2595, *p* = 0.038 in a one-tailed unpaired Student’s *t*-test. The one-tailed test was used to direct statistical power towards detecting a gain-of-function effect previously shown for IGF2). LTF magnitude was not significantly increased by ApILP1 when five pulses of 5HT were administered (5 × 5HT: 0.1883 ± 0.05286, n = 17; 5 × 5HT + ApILP1: 0.2714 ± 0.08836, n = 13). The same peptide caused a reduction in excitability of SNs (control, 4.152 ± 0.1545 [action potentials/mA positive current], n = 16, R^2^ = 0.989; ApILP1, 2.826 ± 0.197, n = 16, R^2^ = 0.9626, *p* < 0.0001 in ANCOVA comparing slopes). Thus, ApILP1 mimicked the effect of IGF2 on neurons even in the absence of appropriate post-translational processing in the bacterial host. We were therefore able to generate this ligand in amounts sufficient for intrahemocoelic injections and test the effect of ApILP1 on the clearance of a glucose load.

First, we verified that *Aplysia* metabolism is controlled by a *bona fide* insulin system by injecting 200–250 g animals with a solution containing glucose (Glc) with or without GSK1838705A (GSK), and monitoring glucose content in the hemolymph during subsequent hours (Fig. 3C). GSK1838705A showed a significant attenuation of glucose absorption in the first hour after injection, possibly reflecting its clearance from the hemolymph at later time points (Glc: 59.8 ± 6.27% initial value, n = 10; Glc + GSK: 86.17 ± 11.59, n = 6; difference 26.91 ± 12; 95% CI 1.162 to 52.66, *p* = 0.0417 in a two-tailed unpaired Student’s *t*-test; one animal was excluded after failing to reduce glucose levels 24 hours after injection). Co-injection of glucose with tolbutamide (Tol), a K_ATP_ channel inhibitor, resulted in lower glucose levels 4–6 h after the injection, although this trend did not reach significance in 200–250 g animals (4 h post-injection, Glc: 16.51 ± 8.137% initial value, n = 10; Glc + Tol: 4.942 ± 2.886, n = 6; difference 11.57 ± 10.88; 95% CI –11.76 to 34.89). In mammals, K_ATP_ blockade mimics the effects of caloric intake, and causes the release of insulin-containing secretory granules from pancreatic β-cells^60^, enabling its use as a proxy for insulin secretion. Further studies involving direct measurements of K_ATP_ currents will be required to confirm whether the response to tolbutamide in *Aplysia* operates via similar mechanisms.

Second, when an injection of glucose into 1–4 g animals was combined with ApILP1 (Fig. 3D), levels of hemolymph glucose were lower 4 h later as compared to animals injected with glucose alone. An even stronger effect was observed in these small animals when glucose was coupled with tolbutamide. One-way ANOVA revealed a significant difference between means (*p* = 0.0029), Dunnett’s multiple comparisons test confirmed a significant decrease in hemolymph glucose levels in response to ApILP1 and tolbutamide (Glc *vs* Glc + ApILP1 difference 206.5 µg glucose/mg protein, 95% CI 37.3 to 375.7, *p* = 0.0188; Glc *vs* Glc + Tol difference 312.6, 95% CI 133.2 to 492.1, *p* = 0.0020). Thus, ApILP1 recapitulated both the metabolic and neurotropic effects of IGF2, suggesting that they are functionally related.

## Discussion

We have demonstrated an unusual combination of effects exerted by IGF2 on *Aplysia* sensory neurons. The peptide promotes both growth of these neurons and their long-term facilitation (Fig. 1), but downregulates their excitability and input resistance (Fig. 2). The downstream molecular targets of IGF2 that mediate these changes in *Aplysia* remain to be established. However, *Aplysia* does possess insulin-like peptides and receptors, which are expressed in both pleural and pedal ganglia (Fig. S2), containing sensory neurons and their synaptic contacts.

A wide range of behavioral plasticity in the *Aplysia* withdrawal reflexes can be accounted for by presynaptic mechanisms, and thus the gain-of-function effects of IGF2 on LTF are likely explained by changes in the SN, consistent with the observed boost in neurite outgrowth. Growth factors are widely implicated in enabling subthreshold learning^61–64^ and promoting neurite outgrowth^65,66^. More surprising is the combination of these effects with reduced excitability of SNs. Sensitization training, for example, increases SN excitability^67^, and the resulting spike broadening has been proposed to contribute to LTF^68^. In principle however, these effects are not necessarily coupled: membrane effects that reduce presynaptic excitability (such as increased outward current) could be accompanied by structural plasticity that facilitates synaptic transmission. Structural plasticity probably explains most of the enhancement of synaptic transmission observed after long-term sensitization training: it has been reported that after repeated 5HT treatment the amount of synaptically active SN varicosities increases 60–75%, which corresponds remarkably well with the extent of synaptic facilitation^69,70^ (e.g. in the present study, 5 × 5HT treatment produced 62–75% facilitation above the pretest). However, the synaptic activity of IGF2-induced sensory neuron varicosities has not been directly measured in this study, and thus it remains a possibility that mechanisms other than structural plasticity account for the enhanced synaptic transmission. It is also a possibility that L7, the motoneuron used in LTF measurements and representing a non-standard postsynaptic target, may influence the gain-of-function effects observed for IGF2 and ApILP1. In either case, the effects of IGF2 and ApILP1 are atypical (but not contradictory), suggesting more refined and subtle functional significance.

**Figure 4:**
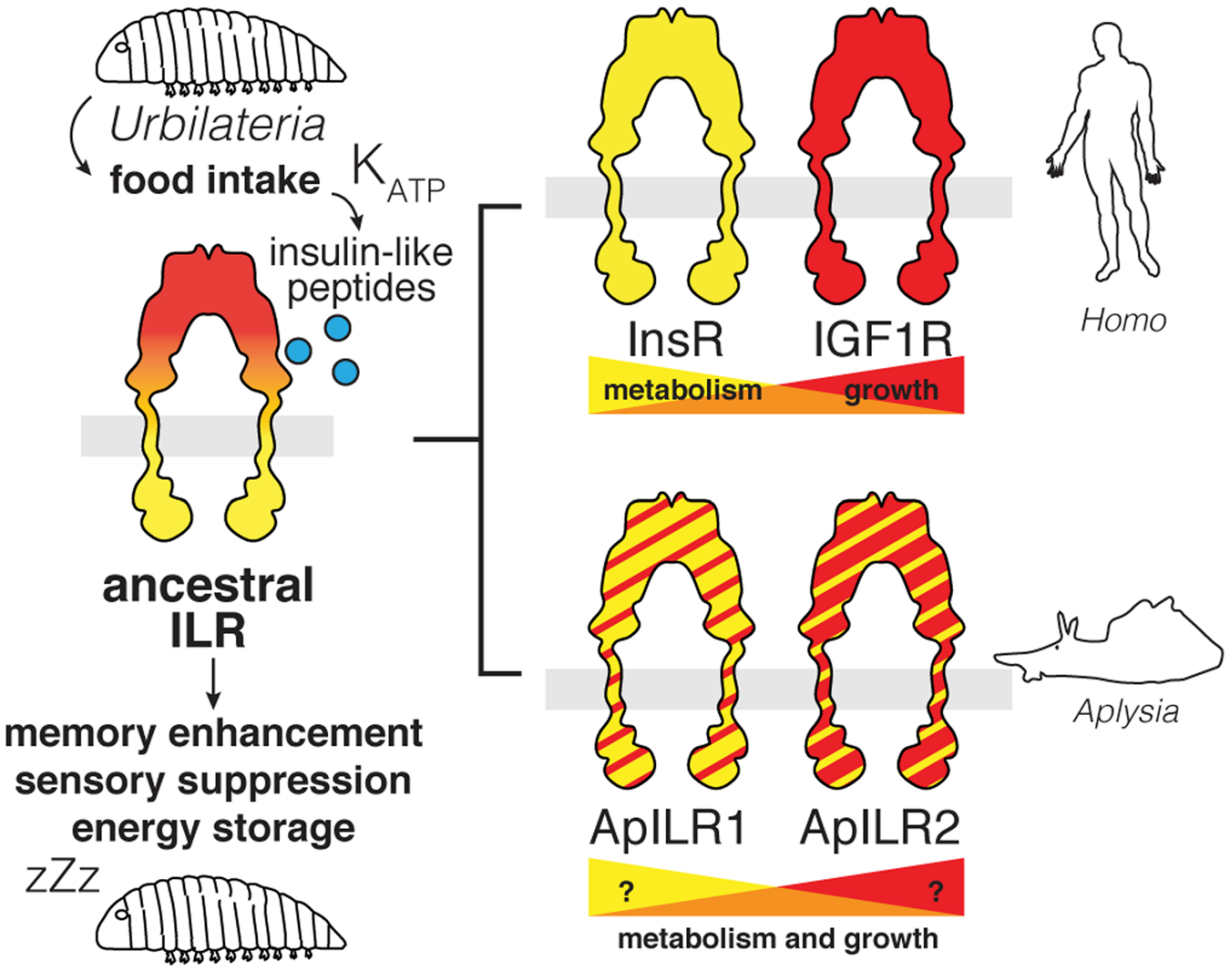
Hypothesis for the evolution of insulin-related functions. An ancestral single-receptor insulin-like system displays both metabolic function (response to feeding, energy storage) and neurotropic function (memory enhancement, sensory suppression). Subspecialization in mammals leads to a partial redistribution of metabolic and growth-related functions between InsR and IGF1R. A parallel duplication of receptors in gastropods does not necessarily reflect the same subspecialization.

Insulin-like systems have been identified in a diverse range of organisms, where they perform a variety of functions ranging from metabolic to neurotropic. While in vertebrates insulin and IGFs are subspecialized into systems with overlapping but distinct functionality, the diversification of invertebrate insulin-like receptors and ligands need not follow the same trend (Fig. 4). Thus, the effects of human IGF2 in *Aplysia* do not reflect a response to IGF2 specifically. Most likely, they represent the activity of an endogenous molluscan insulin-like system. Any function of this system conserved between mammals and gastropods likely reflects ancestral evolutionary drives that shaped a single-receptor insulin-like system in *Urbilateria*, the last common ancestor of these two groups.

Neurotropic and metabolic influences of insulin-like molecules are generally seen as distinct effects. In vertebrates, insulin and its receptor are more involved in energy homeostasis, whereas IGF1 and IGF2 perform additional functions such as neuromodulation. Our results, however, suggest that invertebrate insulin-like systems may have a more unified function. A single peptide replicates the effects of IGF2 on SN excitability and SN-MN synaptic transmission (a neurotropic influence) and at the same time stimulates peripheral glucose absorption (a metabolic influence) (Fig. 3).

We speculate that the combination of neurotropic and metabolic effects of the endogenous *Aplysia* insulin-like system may represent a coordinated response to calorie intake. It has been proposed in a range of systems that long-term memory and active behaviors are energetically costly and require trade-offs^71–74^. It is possible that in *Aplysia*, memory consolidation, and its cellular correlate LTF, are induced as part of a general reallocation of resources in response to feeding. The reduction in sensory neuron excitability may in turn reflect a reciprocal element of this overall strategy. Incidentally, it has been reported^75^ that after feeding, the locomotor activity of *Aplysia* is reduced, as is common in a diverse range of animals. Consistently with this hypothesis, the behavioral effect is significantly attenuated by an injection of GSK1838705A, the inhibitor of InsR/IGF1R in mammals (Fig. S6), suggesting that feeding broadly controls *Aplysia* behavior via insulin-like receptors.

Our results raise important questions regarding the mechanisms of IGF2 enhancement of memory in vertebrates, particularly with regards to M6PR, the unusual receptor required for the IGF2 effects in rodents. Although orthologous molecules are present throughout the animal kingdom and even in non-metazoan eukaryotes, the affinity of this receptor to IGF2 is a recent evolutionary acquisition specific to placental mammals. It is generally thought that the M6PR counteracts IGF2 by “scavenging” it away from insulin-like receptors, particularly IGF1R, and targeting it to lysosomal degradation. How can a ligand enhance memory via a receptor that destroys it? One explanation may be in the engagement of M6PR’s signaling functions unrelated to canonical insulin-like signaling^18–21^. However, based on our data and the degree of functional conservation in the insulin system, one can expect insulin-like peptides to have a complex array of dissociable neuronal effects, and the behavioral outcome of these effects may depend on the precise balance of concentrations and affinities. M6PR might provide secondary means to control this balance in a non-linear way, for instance by differentially coordinating the spatial distribution of IGF2 in various locations across the brain. From this functional evolutionary perspective, *Aplysia* provides a novel and potentially powerful model system to explore ligand-receptor interactions at a point in phylogenetic space where they are not functionally coupled.

## Materials and Methods

### Reagents

Human recombinant IGF2 (0.5 µg/ml) was from R&D. Rapamycin (0.2 µM) was from Calbiochem. All other reagents were from Sigma unless indicated otherwise.

### Cell culture

Sensory neurons were cultured with minor modifications to previously described protocols^76,77^. For isolated SNs, mariculture-raised *Aplysia californica* (60–100 g, *Aplysia* Resource Growout Facility, University of Miami) were anaesthetized by injection of MgCl_2_ (369 mM MgCl_2_, 10 mM Tris-HCl pH 7.6), pleural-pedal ganglia removed and incubated overnight at 22 °C in a solution containing 5U/ml dispase (Gibco) in salt-adjusted L15 (Leibovitz) medium (Sigma; supplemented with 264 mM NaCl, 26 mM MgSO_4_, 27 mM MgCl_2_, 5 mM KCl, 2 mM NaHCO_3_, 11 mM CaCl_2_, 15 mM HEPES, 35 mM glucose, 100 U/ml penicillin, 0.1 mg/ml streptomycin). Ganglia were washed in artificial seawater (ASW; 460 mM NaCl, 55 mM MgCl_2_, 11 mM CaCl_2_, 10 mM KCl, 10 mM Tris-HCl pH 7.6) and desheathed in a 1:1 solution of L15 and ASW. Neurons were extracted from ganglia by pulling with a glass microelectrode. Sensory neurons were identified based on size and location within the ventral sensory cluster in the pleural ganglion. After extraction, neurons were transferred to a glass bottom dish precoated with poly-L-lysine (MW **≥ **300,000 Da; 0.75 mg/ml hydrobromide in 0.1 M sodium borate pH 8.2, ~16 h at room temperature) and laminin from Engelbreth-Holm-Swarm murine sarcoma basement membrane (50 µg/ml in PBS, 2 h at room temperature). Cells were cultured in a humidified atmosphere at 16 °C in full culture medium, i.e. a solution containing 50% salt-adjusted L15, 50% *Aplysia* hemolymph (collected in the spring, pooled from ~10 wild-caught animals and stored at –80 °C), and L-glutamine (2 mM).

Hemolymph concentration was lowered to 10% for experiments involving activity-dependent growth to reduce masking of the effects. For LTF experiments, SNs and MNs were cultured as previously described^46^. MNs were plated first and allowed to attach for 24 h at room temperature, followed by addition of SNs in physical contact to the MN neurites. Electrophysiological recordings were carried out 3–4 days after the extraction of SNs for excitability measurements, or days 4 and 5 for LTF experiments. For growth assays, media were exchanged on day 3, and subsequent growth rates monitored.

### Electrophysiology

Intracellular recording was performed using glass microelectrodes containing 3 M KCl with electrode resistance of 5–12 MΩ. Data were sampled at 1 kHz without filtering. For excitability measurements, sensory neurons in culture or in ganglia were injected with square pulses of positive current (0.1–1 nA) lasting 200 or 500 ms, and number of action potentials elicited was counted. Input resistance was calculated from the linear part of the I/O curve produced by injecting negative current and measuring changes in membrane potential. For LTF experiments, media were exchanged to 1:1 ASW:L15 before impaling motor neurons and hyperpolarizing their membrane to approximately −75 mV. Sensory neurons were then stimulated with an extracellular electrode to elicit a single spike, and EPSPs measured as pre-tests. After recording cells were subjected to 5HT treatments. 5HT was added to culture dishes at a final concentration of 50 µM, and washed out 5 min later by perfusion with 1:1 ASW:L15. For LTF induction, this procedure was repeated five times with an intertrial interval of 15 min. Following the final 5HT washout, media were replaced by freshly prepared full culture medium and incubated as above for 16–20 h. The media were then again exchanged to 1:1 ASW:L15, and the intracellular recordings repeated.

For ganglia recordings, wild-caught *Aplysia californica* (150–250 g, South Coast Bio-Marine) were anaesthetized as above, pleural-pedal ganglia removed and desheathed in a 1:1 solution of MgCl_2_ (369 mM MgCl_2_, 10 mM Tris-HCl pH 7.6) and ASW. Ganglia were perfused with ASW for 30 min, followed by a whole-bath ASW incubation for 90 min, to clear MgCl_2_ prior to experimentation. Drug administration was not blinded, however data analysis was performed by an operator blind to experimental conditions.

### Sholl analysis

For morphological analysis of neurite outgrowth, images of each neuron grown in culture were taken on multiple days. Images were processed in Adobe Photoshop using the *find edges* command and manually traced to remove debris from the image by an operator blind to the experimental procedure. After thresholding, images were analyzed in Fiji using the *Sholl analysis* plugin. The software generates 20 concentric circles around the manually defined soma and plots intersecting density against each circle number. Areas under the resultant curves (AUCs) were then calculated for each image (Fig. 2). These values were highly variable, since neurons can lose or retain neurites of variable length upon plating, which strongly affected their morphology on subsequent days. However, neurite growth rates in proportion to neurite arborization already present (“growth rates per neurite”) were highly consistent between cells. For this reason, growth rates were calculated by normalizing daily changes in these values to AUC on the preceding day.

### Sequence analysis

The sequences of insulin-like receptors were found using BLASTp against protein database and the translated BLAST against transcriptomic nucleotide sequences by using human and *Aplysia* genes as queries. Cephalopoda was used as an outgroup for Gastropoda, Platyhelminthes was used as an outgroup for Mollusca, and Cnidaria as an outgroup for Bilateria. Full-length protein sequences were aligned with MAFFT (v. 7.310) using automatically selected parameters (L-INS-i). Dataset was optimized using MaxAlign to increase the number of gap-free sites. A total of 42 sequences and 604 conserved sites were used for phylogenetic estimation. The maximum likelihood (ML) trees were built using the neighbor-joining method and the Jones-Taylor-Thornton substitution model, followed by bootstrap resampling with 1000 repetitions.

### Glucose absorption

200–250 g animals were injected with a solution containing glucose (100 mg/kg body weight) with or without tolbutamide (27 mg/kg body weight) or GSK1838705A (0.5 μmol/kg body weight) or vehicle (DMSO). ~1 ml samples of hemolymph were collected 15 min and every hour after the injection. Samples were cleared by centrifugation, followed by measurements of total glucose and total protein. Glucose was measured using the colorimetric glucose oxidase assay (Sigma). Protein was measured using the BCA assay (Thermo Fisher). Glucose measurements were standardized to protein amounts to account for dilution. Glucose levels 15 min after the injection were taken as 100%, and subsequent within-animal levels expressed relative to this measurement. 1–4 g animals were injected with a solution containing glucose (500 mg/kg body weight) with or without ApILP1 (5 mg/kg body weight) or tolbutamide (27 mg/kg body weight). Hemolymph was collected 4 h later, and glucose levels standardized to protein content.

### RNA Isolation, cDNA Synthesis, PCR and cloning

RNA was isolated and purified using QIAGEN RNeasy mini kit columns (QIAGEN). cDNA was synthesized using Superscript IV CellsDirect cDNA synthesis reagents (Invitrogen). Residual DNA was hydrolyzed with Turbo DNase (Thermo Fisher). Control reactions without reverse transcriptase were used to monitor for genomic DNA contamination. Primers, amplification targets and expected product sizes are given in Table 1. PCR was performed using Phusion DNA polymerase (New England Biolabs), according to manufacturer’s instructions (30 s primer anneal at 58 °C, 30 s amplification at 72 °C, 10 s denaturation at 98 °C, 25 cycles). All PCR products matched their expected sizes. For ApILP1 expression, full-length ApILP1 was inserted into the pGEX4T-1 vector with an N-terminal GST affinity tag and a C-terminal FLAG tag to verify expression of the full-length protein.

**Table 1:** PCR primers and targets used in this study.

Name	Accession no.	Forward primer (5′–3′)	Reverse primer (5′–3′)	Expected product size, bp
GAPDH	NP_001267755.1	CTCTGAGGGTGCTTTGAAGG	GTTGTCGTTGAGGGCAATTC	124
ApILR1	XP_005096967.1	CCTCCAGGCTGTTCACATCA	GTCTCCAAGTGCCCAGACAA	123
ApILR2	XP_012935847.1	GAAACAGGCAGAGACGATGG	GGCCACGCTGATATGTGTCT	189
ApILP1	NM_001204695.1	ACCGAGACTGCTTCGTTTGT	ACCGAGACTGCTTCGTTTGT	796
ApILP2	GBBG01039292.1	GAGTGAGAGAACAGAAGGTGGA	TAAAGTAGTGCCCGGAGTGC	765
ApILP3	NM_001204686.1	ATCTCCGGTGAGCAGTGTTG	GCGGGTATCGCTGGTATGAA	754
ApILP4	GBBG01097053.1	ATTTGCGAAGGGTTGTGTGC	CCAAATTGGTTACATGCCCCT	741
ApILP5	NM_001204574.1	GTGTGAGCTGTGTGTCTGGA	AACCCCAGCAGAAACCGAAA	733

### Protein expression and purification

Bacterial cultures (Rosetta 2) were grown to OD600 = 0.7, followed by induction with 1 mM IPTG for 2 h at 37 °C. Cells were washed in PBS and lysed in a buffer containing HEPES (25 mM pH 7.4), NaCl (150 mM), glycerol (10% v/v), lysozyme (0.5 mg/ml), DNase, and Triton X-100 (0.5% v/v), followed by sonication. Lysates were cleared by centrifugation at 100,000 *g* for 1 h. GST-ApILP1 was purified by incubating with GSH-agarose for 2 h, followed by thrombin cleavage.

### Locomotor activity

100–150 g animals were housed in colanders, and their behavior recorded using a camera positioned above the aquarium. Recordings were performed only during light hours of the day, and no significant differences in average hourly activity were seen throughout the day. Following food deprivation lasting 1 week, animals were fed *ad libitum* with roasted nori seaweed for 4 h. Behavior was monitored immediately after feeding and on the subsequent day and compared to the corresponding periods on days preceding feeding. Animals received four injections of GSK1838705A (0.5 μmol/kg body weight) or vehicle (DMSO) before and after feeding and at the same times on the subsequent day. Average hourly activity was calculated by manually scoring videos in JWatcher (time-lapse, 12 × speed) according to the following rule: 0 – animal is static; 1 – animal moves while attached to the colander (“seeking”); 2 – animal is actively moving. Videos were scored by an operator blind to experimental conditions.

### Statistical analyses

All data were analyzed using GraphPad Prism. All data are shown as means ± SEM. Normal distribution was verified using the D’Agostino-Pearson test. In most cases, statistical significance is assessed using two-tailed Student’s *t*-tests unless indicated otherwise. For linear regression, slopes were compared using ANCOVA. For multiple comparisons, an ordinary one-way ANOVA was followed by Dunnett’s post-hoc tests to compare multiple treatments to a single control.

### Ethical approval and informed consent

This study does not employ humans or human samples, other vertebrates or higher invertebrates.

## Supplementary information

Supplementary Materials

## Data Availability

The datasets generated during and/or analyzed during the current study are available from the corresponding author on reasonable request.
