# Supplementary material for: Neurotropic and modulatory effects of insulin-like growth factor II in Aplysia
Source: Sci Rep. 2019 Oct 7;9:14379. doi: 10.1038/s41598-019-50923-5 (PMC6779898; doi:10.1038/s41598-019-50923-5)
Supplement: Supplementary file 1 — Supplementary Materials [file 41598_2019_50923_MOESM1_ESM.docx]

**Supplementary Materials**

**Neurotropic and modulatory effects of insulin-like growth factor II in *Aplysia***

Nikolay Vadimovich Kukushkin^1^, Sidney Paulina Williams^1^, and Thomas James Carew^1*^

**Supplemental Figure 1: Activity-dependent neurite outgrowth**

Related to Figure 2

**
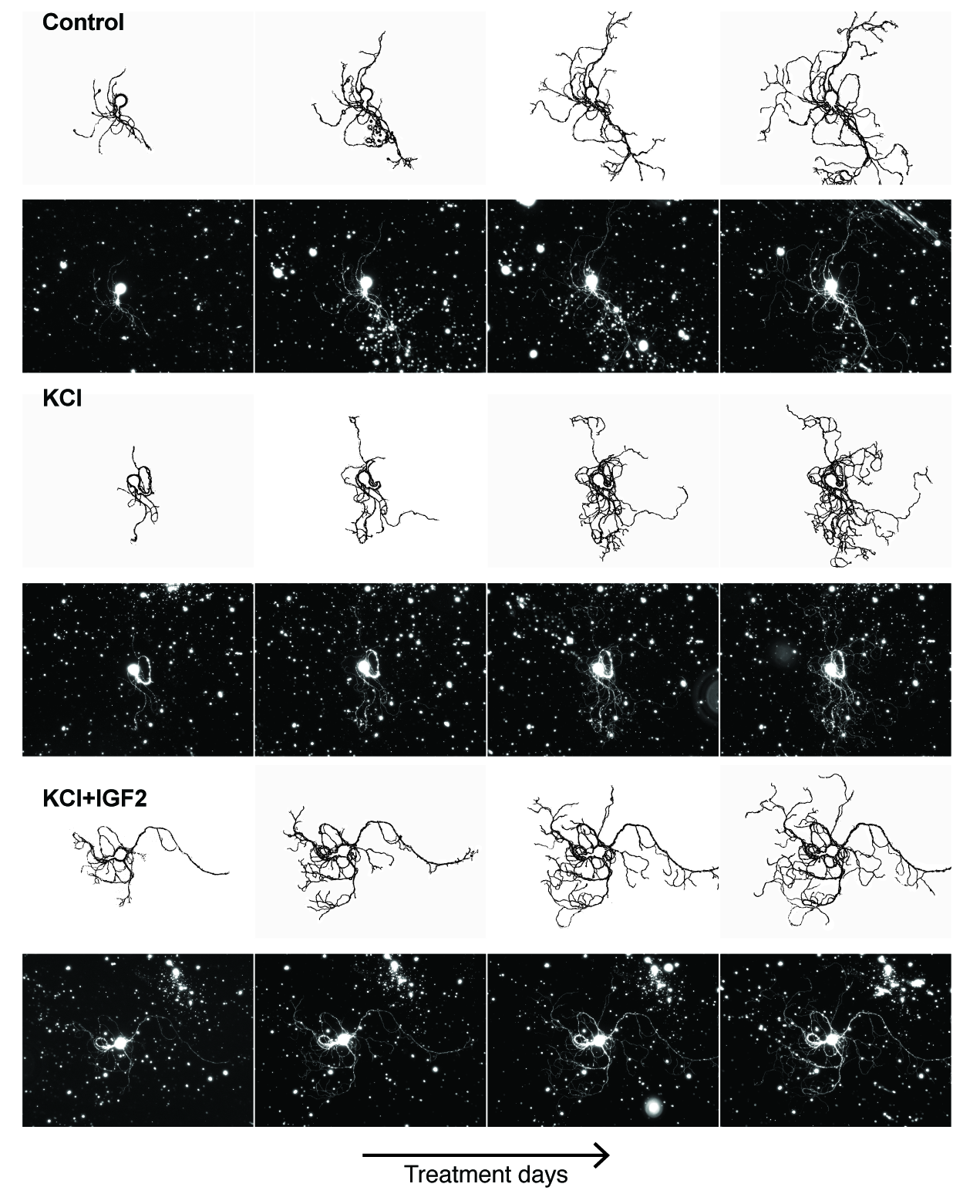
**

Supplemental Figure 1. Representative micrographs of sensory neuron outgrowth as processed by Sholl analysis in Figure 2B. Upper rows, processed micrographs. Lower rows, raw images.

**Supplemental Figure 2: Aplysia insulin-like receptors and peptides**

Related to Figure 3


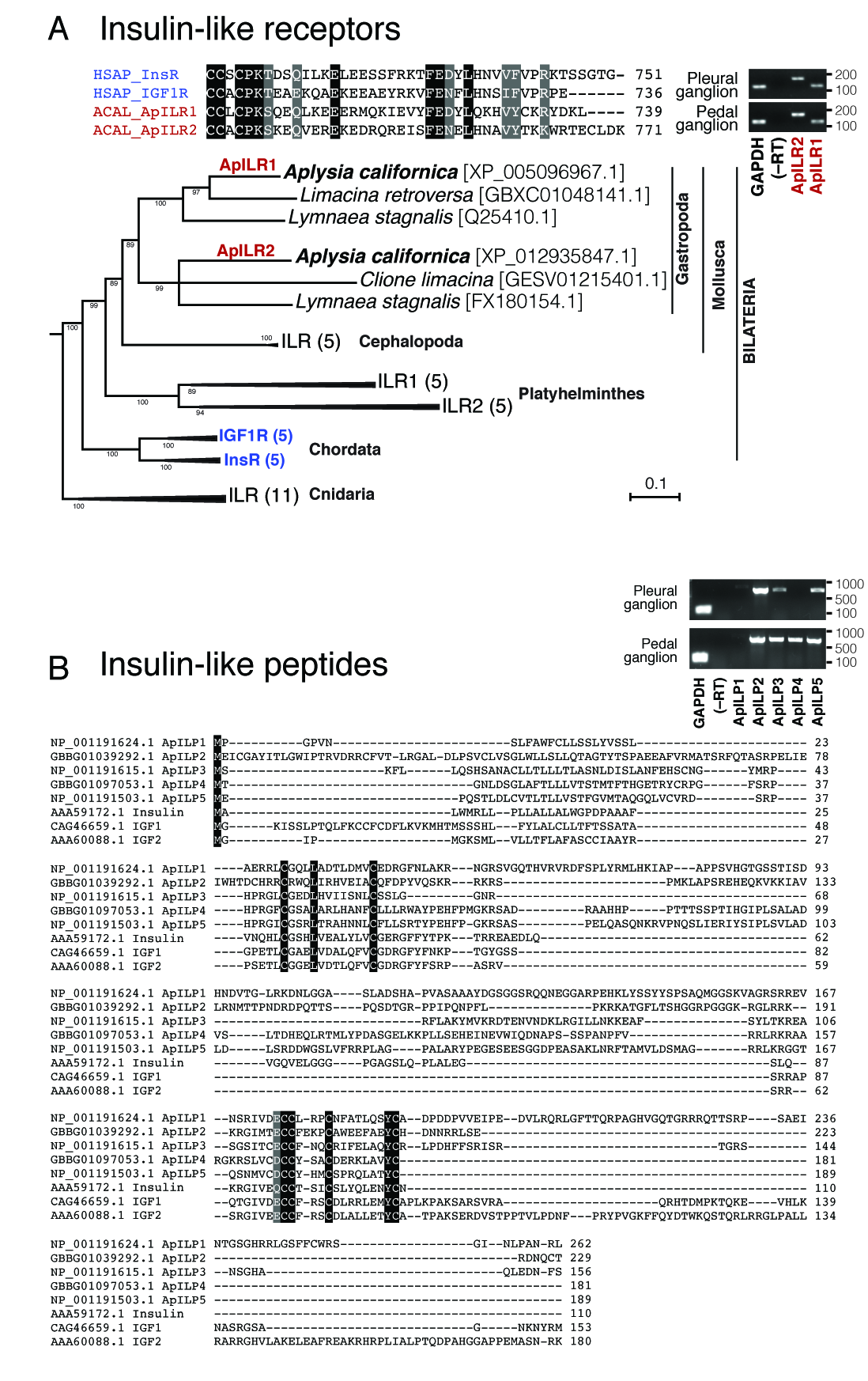


Supplemental Figure 2. A. Top left, alignment of amino acid sequences corresponding to substrate-binding ɑC-termini of human (HSAP) and *Aplysia* (ACAL) insulin-like receptors. InsR, insulin receptor. IGF1R, IGF1 receptor. ApILR1–2, *Aplysia* insulin-like receptors. Top right, ApILR1–2 are expressed in both pleural and pedal ganglia as confirmed by RT-PCR. GAPDH amplification in samples with or without reverse transcriptase (RT) was used as a control. Bottom, cladogram representing maximum-likelihood estimation of evolutionary relationships between vertebrate and gastropod insulin-like receptors (accession numbers shown in parentheses). Numbers indicate bootstrap support. B. Right, expression of insulin-like peptides (termed ApILP1–5) in *Aplysia* pleural/pedal ganglia. Sequences were identified by similarity with human insulin/IGFs. RNA was extracted from ganglia, and expression monitored by RT-PCR. GAPDH amplification in samples with or without reverse transcriptase (RT) was used as a control. Bottom, alignment of amino acid sequences of ApILP1–5 with human insulin, IGF1 and IGF2 precursors.

**Supplemental Figure 3: Aplysia insulin-like receptor phylogenetic modeling**

Related to Supplemental Figure 2


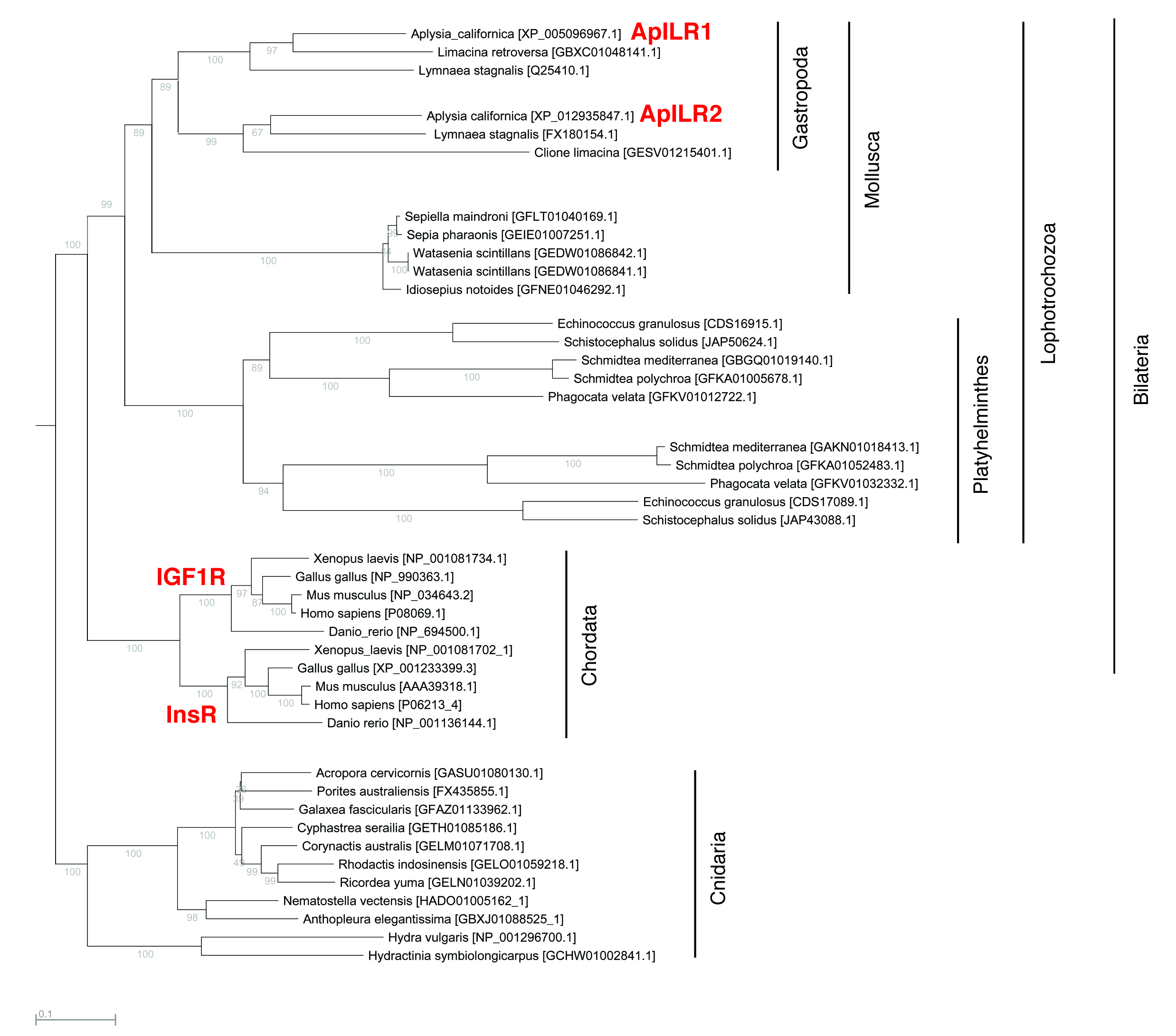


Supplemental Figure 3. Full cladogram of shown in Supplemental Figure 1, representing maximum-likelihood estimation of evolutionary relationships between vertebrate and gastropod insulin-like receptors (accession numbers shown in parentheses). Numbers indicate bootstrap support.

**Supplemental Figure 4: Aplysia insulin-like receptors and peptides**

Related to Supplemental Figure 2


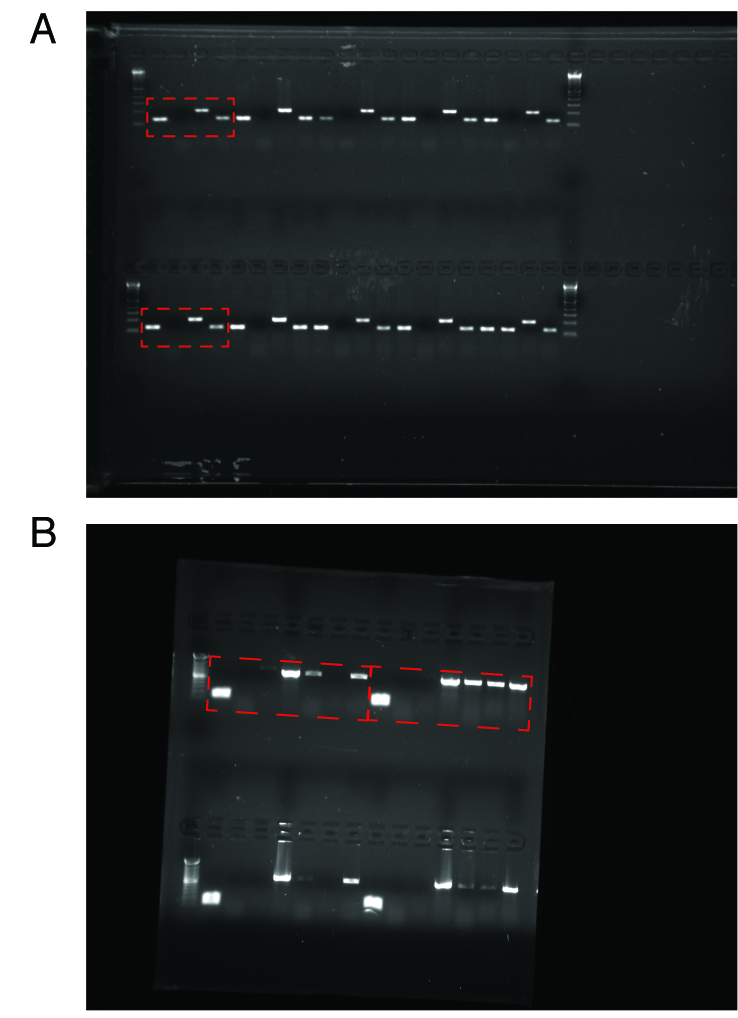


Supplemental Figure 4. Unprocessed gel images used in Supplemental Figure 1. Regions cropped and annotated in Supplemental Figure 1 are framed in red. A, insulin-like receptors. B, insulin-like peptides.

**Supplemental Figure 5: Rapamycin blocks IGF2 effect on excitability of cultured neurons**

Related to Figure 2

Supplemental Figure 5. Left, excitability of sensory neurons was monitored by intracellular depolarizing current injections. Asterisk represents *p*<0.05 in a two-tailed unpaired Student’s *t*-test (1.4 nA current injection; vehicle: 5.297 ± 0.3853 action potentials, n=37 *vs* IGF2 + vehicle: 4 ± 0.3581, n=39, difference 1.297 ± 0.5253, 95% CI 0.2506 to 2.344). Larger than usual sample sizes are due to pooling of IGF2+vehicle data from multiple experiments during protocol optimization.

**Supplemental Figure 6: Feeding suppresses Aplysia locomotor activity in an GSK1838705A-sensitive fashion**

Related to Discussion


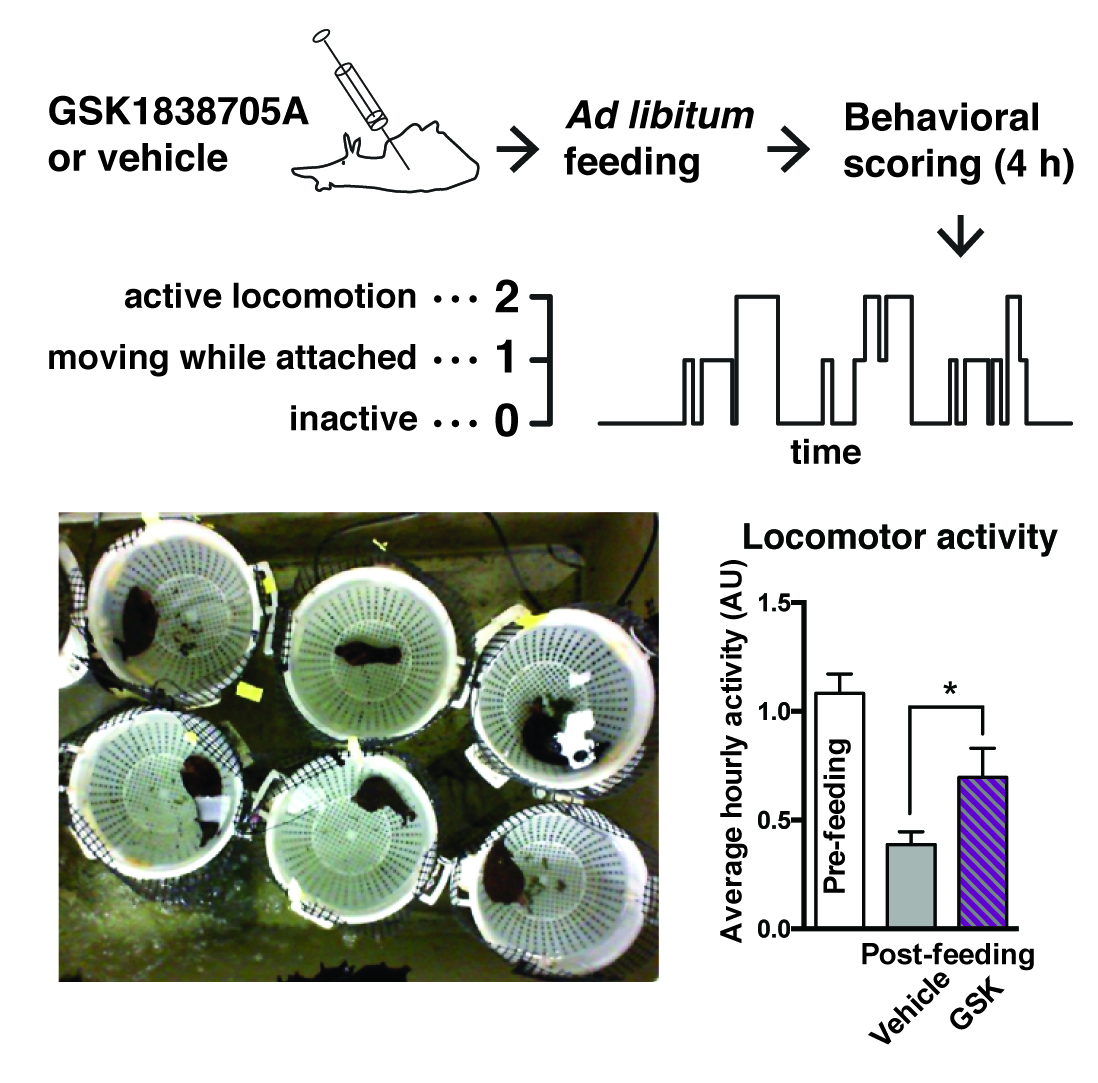


Supplemental Figure 6. *Aplysia* behavior was recorded during light hours using a top-positioned camera. Animals (n=6) were injected with either vehicle or GSK1838705A (0.5 µmol/kg body weight) immediately before feeding, their locomotor activity scored manually over 4-hour periods following the injections, and average hourly activity expressed in arbitrary units as shown. Behavior during the same hours on days preceding feeding was used as control. Columns are means ±SEM. Vehicle: 0.3876 ± 0.0588 [arbitrary units of locomotor activity], n=12; GSK1838705A: 0.6967 ± 0.1334, n=10; difference 0.3091 ± 0.1373; 95% CI 0.02263 to 0.5955; *, *p*=0.036 in a two-tailed unpaired Student’s *t*-test.
